# A qualitative study of the experience of CenteringPregnancy group prenatal care for physicians

**DOI:** 10.1186/1471-2393-13-S1-S6

**Published:** 2013-01-31

**Authors:** Deborah A McNeil, Monica Vekved, Siobhan M Dolan, Jodi Siever, Sarah Horn, Suzanne C Tough

**Affiliations:** 1 Public Health Innovation and Decision Support, Population and Public Health, Alberta Health Services, 10101 Southport Rd SW, Calgary, Alberta, T2W 3N2, Canada; 2 Faculty of Nursing, University of Calgary, 2500 University Drive NW, Calgary, Alberta, T2N 1N4, Canada; 3 Department of Paediatrics, Faculty of Medicine, University of Calgary, 2888 Shaganappi Trail NW, Calgary, Alberta, T3B 6A8, Canada; 4 Department of Obstetrics and Gynecology and Women’s Health, Albert Einstein College of Medicine of Yeshiva University & Montefiore Medical Center, Jack and Pearl Resnick Campus, 1300 Morris Park Avenue, Bronx, New York, 10461, USA; 5 Department Community Health Sciences, Faculty of Medicine, University of Calgary, 3280 Hospital Drive NW, Calgary, Alberta, T2N 4Z6, Canada

## Abstract

**Background:**

This study sought to understand the central meaning of the experience of group prenatal care for physicians who were involved in providing CenteringPregnancy through a maternity clinic in Calgary, Canada.

**Method:**

The study followed the phenomenological qualitative tradition. Three physicians involved in group prenatal care participated in a one-on-one interview between November and December 2009. Two physicians participated in verification sessions. Interviews followed an open ended general guide and were audio recorded and transcribed. The purpose of the analysis was to identify meaning themes and the core meaning experienced by the physicians.

**Results:**

Six themes emerged: (1) *having a greater exchange of information,* (2) *getting to knowing,* (3) *seeing women get to know and support each other,* (4) *sharing ownership of care,* (5) *having more time, and* (6) e*xperiencing enjoyment and satisfaction in providing care*. These themes contributed to the core meaning for physicians of *“providing richer care.”*

**Conclusions:**

Physicians perceived providing better care and a better professional experience through CenteringPregnancy compared to their experience of individual prenatal care. Thus, CenteringPregnancy could improve work place satisfaction, increase retention of providers in maternity care, and improve health care for women.

## Introduction

In 2006, approximately 90% of pregnant women in Canada received prenatal care through one-on-one visits with a physician (58% from an obstetrician/gynaecologist and 34% from a family physician) with 6% receiving care from a midwife [1]. In addition to individual visits with their prenatal care provider, covered financially under the provincial health insurance plan, some Canadian women also pay a fee to attend childbirth education classes. Group prenatal care, which is gaining momentum in the United States and elsewhere, allows women to experience medical care and childbirth education simultaneously in a group setting [2].

Research evidence suggests that women who participate in group prenatal care, specifically the CenteringPregnancy model (hereafter referred to as CenteringPregnancy), have improved prenatal knowledge, greater satisfaction with care, a higher likelihood of having an adequate number of prenatal visits, decreased risk of preterm birth, and a greater readiness for delivery and baby care, compared to women who receive individual prenatal care [3,4]. Qualitative evidence indicates that through CenteringPregnancy, women receive more than they realize they need, in terms of information, support, connection with other women and their providers, normalization and identification with other women, efficiency of care, and ownership of care [5-8]. No previous studies have examined the experience of prenatal care providers involved in CenteringPregnancy. The objective of this study was to understand the central meaning or core experience of providing CenteringPregnancy among family physicians in a community based maternity clinic.

The declining rate of North American family physicians who are involved in providing primary care obstetrics [9-12] and the relatively low job satisfaction among obstetricians [13,14] is reducing the capacity of the future workforce to handle the care of pregnant and delivering women [15]. In particular, poor job satisfaction is associated with early retirement [15,16], cutting back on hours [16], and higher turnover [17]. Physician satisfaction may also be related to patients’ ratings of care [18,19], patients’ tendency to adhere to medical recommendations [20], and the likelihood of patients continuing to receive care from the same physician [18]. Understanding the physician’s professional experience of a particular model of care, such as CenteringPregnancy, could aid medical associations and health care systems in developing strategies to improve job satisfaction among those practicing obstetrics and increase the involvement of family physicians and residents in primary care obstetrics. The following research question guided this study. What is the experience of CenteringPregnancy from the physician’s perspective?

## Methods

### Study design

A qualitative research approach to address the research question provided a method for in-depth exploration of this group of physicians’ experiences that was context specific and that would not be possible using surveys or questionnaires. Common perceptions and meaning were elucidated through interview and analytic approaches focusing on study participants’ language. This study used phenomenology and Heidegger’s approach to inquiry, in particular, to study the family physician experience of CenteringPregnancy [21]. The basic premise is that cultural groups with common experience share common meanings about a phenomenon that provide insight into their experience, and that their language can be used to identify the meaning [21]. This qualitative research approach moves beyond describing an experience to providing an understanding of the meaning of the experience of the cultural group being studied. CenteringPregnancy was the phenomenon being studied and the meaning of the experience from the physician perspective was sought. This study of physicians was complemented by similar qualitative studies from both the women’s experiences [5] and the child birth educators’ perceptions.

### Model of care

This particular CenteringPregnancy program was led by family physicians that co-facilitated group sessions with a perinatal educator. The family physicians were part of a group practice that provides maternity care to women with low risk pregnancies in Calgary, Canada and did not receive additional remuneration for providing CenteringPregnancy. Women receiving their prenatal care through CenteringPregnancy were part of a cohort study of CenteringPregnancy and did not pay additionally to participate in the group sessions [5]. The physicians and educators received two days of training in the CenteringPregnancy model through the Centering Healthcare Institute, and one physician sought advanced training [22]. Physicians from the group maternity practice (some of whom provided the CenteringPregnancy program and some who did not) attended the deliveries of the infants of the women in the program.

All key components of CenteringPregnancy were followed in this study. Pregnant women received prenatal care over 10 two-hour sessions in groups of 8 to 12 women of similar gestational ages [23]. Group sessions started early in the second trimester of pregnancy [23]. During each session, women underwent an individual physical assessment in the group space with the family physician, took their own blood pressure and weight, participated in facilitated group discussions, and interacted with the physician, perinatal educator, and each other [23]. While each session had an overarching plan to discuss relevant pregnancy, childbirth, and parenting topics, the session was led by providers in a facilitative manner enabling the group to direct and contribute to the content [23].

### Recruitment, sample, and data collection

At the time of this study, three family physicians were offering CenteringPregnancy. A research assistant invited each of these physicians to participate in a one-on-one interview about their experience with CenteringPregnancy. Between November and December 2009, one of two interviewers (DAM or MV, neither members of the health care team providing CenteringPregnancy) met with each participant at a mutually convenient location. The interviews ranged from 20 to 40 minutes, were audio recorded, and transcribed verbatim without names. Table 1 outlines the interview guide used. All participants provided written informed consent and were referred to by a study identification number for analysis. The study was approved by the University of Calgary Conjoint Health Research Ethics Board.

**Table 1:** Interview guide

Central interview question	What was it like for you to provide this type of care?
**Additional questions**	What was the best thing about providing group prenatal care? What was the worst thing about it? What about this experience went as expected? What about this experience did not go as expected?

**Probes**	Can you tell me more about what that was like for you or meant to you?

### Data analysis

Each investigator on the study team wrote a description of their personal experience with prenatal care and identified the potential influence of their experience on the analysis to address reflexivity (the investigators potential influence on the analysis) [24]. Each investigator then read the transcripts independently multiple times to facilitate dependability [24]. In the coding process, investigators highlighted and noted statements that identified meaning related to the CenteringPregnancy experience, grouped these statements into “meaning units” or themes, and composed a description. As a group, the investigative team explored possible meanings and divergent perspectives, meeting on a regular basis to reach consensus on meaning themes and to develop an overall description of the “essence” or core of the experience. During this process, the team continually returned to the text and associated meaning statements to also facilitate dependability [24]. To address confirmability, verification interviews were held with two of the three physicians approximately fifteen months after the original interviews [24]. At these sessions, one of the investigators, DAM, shared preliminary findings and requested feedback on the themes and core experience that emerged from the analysis. Each session lasted approximately 60 minutes.

## Results

All three physicians were invited and participated in the initial one-on-one interviews. Two physicians participated in confirmation sessions. All participants were female. Two had been providing obstetrical care for less than 5 years and one for over 20 years.

**Figure 1:**
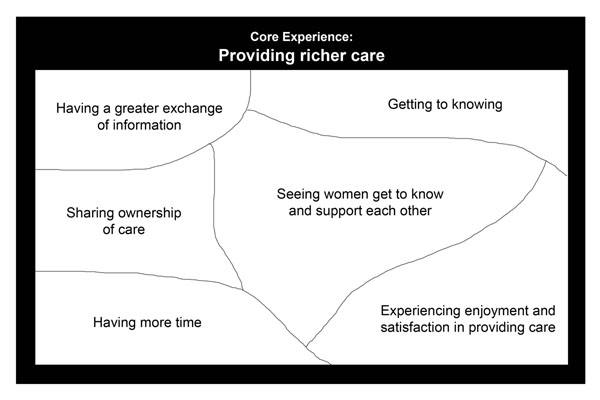
The experience of CenteringPregnancy for physicians

During analysis, six themes emerged that described the meaning of the experience for the physicians providing CenteringPregnancy and contributed to understanding their core experience (Figure 1). The confirmatory sessions validated the themes and the core experience. These are presented in the following sections using exemplars from the participants.

### Theme: having a greater exchange of information

Physicians experienced more two-way communication with the women in their care. They could provide more detailed explanations in CenteringPregnancy than in individual care because *“to have a fifteen minute discussion with ten people is a lot better than…ten four minute discussions”* (Physician 2). Physicians also received more information from the women since they *“got to know a little bit more personally about [the women]…their home situation or their concerns about pregnancy”* (Physician 3). Group discussions allowed topics to be explored more thoroughly. *“Like when we talked about H1N1… everybody can bring up the myths that they’ve heard or the good information that they’ve heard and we can discuss them as a group”* (Physician 2).

### Theme: getting to knowing

Physicians got to *“know more about [women] as people…as opposed to…patients”* (Physician 1) through hearing their stories, backgrounds, struggles, and worries. They also became more perceptive in their interactions with women. *“I sort of know…when they ask a question what they’re actually trying to get at. Because sometimes they ask you a question hoping that you’ll give them more information than what they’ve actually asked”* (Physician 1). Overall, physicians developed a relationship with patients where *“there’s a deeper trust that builds”* (Physician 1), and these enhanced relationships improved the care experience. *“When you have a better relationship*, *you feel like you’re providing better care because I think there’s less likely to be a hidden agenda or worries that the patient has that they don’t bring forward to you”* (Physician 3).

### Theme: seeing women get to know and support each other

Physicians observed women get to know each other and the relationships that developed between women. *“It feels like a rich experience that the women and their partners are getting…just*, *seeing connections…they’re exchanging emails and keeping in touch”* (Physician 2). Women shared information with each other, and physicians saw that women benefitted from this. *“When they’re getting input from the other group members…I think they come out of the program having a more complete understanding of labour and birth*, *being new parents…than they would get if they’re only doing the doctor’s visit as much as we try to do what we do”* (Physician 2).

### Theme: sharing ownership of care

The physicians perceived that they shared ownership of care with women and *“their opinion and what they’ve read is valued in group as much as what I say”* (Physician 1). The group sessions provided opportunities for the physicians to draw upon women’s knowledge to communicate critical information to the group. For instance, one woman shared a *“detailed birth story that just highlighted so many of the things that I would want them to know and understand about birth but coming from someone that they saw as their peer…a different source besides the doctors or nurses”* (Physician 2). As physicians shared ownership of care with women, they saw women become *“confident…about what they had learned”* (Physician 1) and proficient in participating in their care as “*the women themselves got better and better at doing their blood pressure checks and their urine dips*” (Physician 1).

Physicians also shared ownership of care with the educators and *“didn’t feel like…everything was on me”* (Physician 3). The physicians saw the educators filling a different but complementary role. *“They’ve got experience both in group facilitation and in prenatal education so… their experience just has really added a huge component”* (Physician 2). The physicians greatly appreciated the contribution of the educators to the care and thought they were *“worth their weight in gold”* (Physician 1).

### Theme: having more time

The physicians had more time in CenteringPregnancy than with individual care because time was used efficiently. *“When I’m doing one-on-one care…I have more time constraints…I can’t impart everything I’ve learned from twenty years of delivering babies in…five seven minute visits…but*, *I can get more of that across in…all their two hour groups”* (Physician 2). Having more time in CenteringPregnancy allowed for the development of more trust in the physician-patient relationship and facilitated physicians*“[getting] to know people better”* (Physician 1), women feeling more comfortable asking questions, and a greater exchange of information as *“everything gets covered*, *and it doesn’t feel rushed”* (Physician 3). In essence, the physicians had time to understand women’s needs and provided more comprehensive care. *“In our regular clinic…sometimes we’re kind of rushed and moving pretty quickly and so [I like] to just feel like we can sit down and get in depth with people…I like that…I’d rather have a thick novel than a one paragraph of a magazine article”* (Physician 2).

### Theme: experiencing enjoyment and satisfaction in providing care

Physicians found that being involved in CenteringPregnancy *“was a really positive experience. It was very fun…it’s not stressful”* (Physician 1). Physicians appreciated that care was more efficient and less repetitive. *“Some of the things…that you say a hundred times a day*, *explaining group B strep swab… it’s nice to only do it once in a group care setting”* (Physician 1). Physicians enjoyed seeing connections and support develop between women and couples as well as increased confidence and knowledge. Physicians noted that they *“really enjoyed”* (Physician 3 & Physician 2) CenteringPregnancy because of the improved relationships they had with patients and the higher quality of care they felt they could contribute.

### The core of the experience: providing richer care

Through CenteringPregnancy, physicians experienced providing richer care. *“[With CenteringPregnancy] it feels like we’re able to provide a much…richer quality of care to the patients…sometimes I feel like when I go back to giving my regular care to the other patients*, *it’s like*, *oh*, *it’s not fair*, *you’re not getting as much as the other ones are”* (Physician 2). Care was perceived to be of higher quality because physicians had more time, developed better relationships, had more two-way communication with women, and developed a partnership with the educators. This type of care led to a greater sense of satisfaction with their practice.

There was an acknowledgement that this better care was not just a result of the physicians’ contributions but also due to the relationships and support that occurred between the women. The experience of providing richer care was reinforced when providers saw the benefit of CenteringPregnancy for their patients.

*“I could sit and have a more in depth discussion with people and when they’re getting input from the other group members, so much richer... You can see…the patients come into labour…kind of getting it more completely about what’s going to happen in labour and just seeming more kind of confident, prepared and ready to go through the labour process”* (Physician 2).

## Discussion

The key finding of this research is that physicians who provided CenteringPregnancy were “providing richer care”. Key words used to describe the experience (i.e., “richer,” “more”) suggest physicians are describing their experience partially through comparison to individual prenatal care. Compared to the individual care experience, CenteringPregnancy enabled physicians to have more time, gather and offer more information, and provide better care for their patients. Physicians were highly satisfied providing care through CenteringPregnancy.

These results complement findings of the women’s experience [5]. Where physicians provided richer care, women received more than they realized they needed, having both their conscious and subconscious needs met [5]. Both physicians and women noted an enhanced physician-patient relationship and an effective use of time [5]. While physicians experienced a greater exchange of information, women reported learning meaningful information, and where physicians shared ownership of care, women actively participated and felt they took ownership of their care [5]. Physicians also witnessed the connections that women experienced with other women and the support they received [5].

The findings of this study have implications for the care of pregnant women. Job satisfaction is thought to be associated with personality, aspects of the work, and the work environment [25]. Among physicians, factors such as caring for patients and a sense of accomplishment have a strong association with job satisfaction [14,25,26]. By enabling richer, higher quality care, CenteringPregnancy enhanced job satisfaction for physicians and may have provided a sense of accomplishment to physicians beyond that experienced in individual prenatal care.

With substantial proportions of obstetricians reporting inadequate time with patients [14], it is noteworthy that physicians perceived they had more time with patients in CenteringPregnancy. Physicians perceived ability to provide quality care is related in part to adequate time with patients [14], and adequate time with patients is associated with lower rates of burnout [27] and greater satisfaction among physicians [28,29]. Thus, more time with patients in CenteringPregnancy may contribute to physicians’ perception of providing higher quality of care as well as greater enjoyment and satisfaction in their work. Furthermore, by using time more effectively and reducing repetitive tasks for physicians, CenteringPregnancy enabled more varied, comprehensive care (e.g., psychosocial aspects of pregnancy) that address important needs among women. Having time to be involved in less routine and more “creative” aspects of care might also lead to greater job satisfaction for physicians [25].

Research suggests opportunities for better communication between physicians and patients may increase patient satisfaction with care and subsequent adherence to medical recommendations [30-32]. Research on CenteringPregnancy indicates that this model of care contributes to greater patient satisfaction [3,4]. This could be attributed to better communication through more time with the physician [33], support for psychosocial aspects of health [32,34], and the participatory nature of the care experience [35].

Physicians who provide prenatal care can use information from this study to make decisions about selecting their model of care, and family medicine residents may find this information helpful in deciding whether to become involved in primary care obstetrics. For family physicians, CenteringPregnancy could enable the incorporation of obstetrics in their practices, providing them with opportunities for increased variety in their work while caring for a generally healthy population who typically have good outcomes [36,37]. In this particular program, the family physicians were part of a group maternity practice. CenteringPregnancy embedded in a group maternity practice could offer unique benefits (e.g., scheduled call times, more work-life balance) that improve job satisfaction [13,15,36,37]. Developing health care systems to support innovative and effective models of prenatal care, such as CenteringPregnancy, may be one strategy to improve job satisfaction among physicians, assist with recruiting family medicine graduates to primary care obstetrics, and retain physicians who practice obstetrics.

### Strengths, limitations and further research

To maintain scientific rigor, this study followed qualitative research standards, describing processes put in place to facilitate reader consideration of credibility and transferability to their setting and context [24]. The findings of this research are based on the perspectives of a small sample of family physicians agreeable to working in this model of care. Further research with other and larger groups of family physicians could corroborate these findings and yield more generalized conclusions. Similar research with midwives and obstetricians would identify if findings are consistent among other types of practitioners. Furthermore, as more maternity care practices use CenteringPregnancy as a model, there is an opportunity to quantitatively measure job satisfaction among those providing CenteringPregnancy compared with those providing individual prenatal care.

## Conclusion

CenteringPregnancy represents a different approach to caring for pregnant women. Physicians practicing this model of care have the sense that they are providing richer care to their patients and experience satisfaction with this type of care. Physicians frustrated with the limitations and time pressures of individual prenatal care may find CenteringPregnancy to be a better experience professionally and a way of providing better care to women. The positive care experience for both physicians and women involved in CenteringPregnancy could change the way health systems plan prenatal care services.

## Competing interests

The authors declare that they have no competing interests.

## Authors’ contributions

DAM and SCT conceived and designed the study. DAM and MV acquired the data. All authors contributed to the analysis and interpretation of data. DAM and MV drafted the manuscript, and SMD, JS, SH, and SCT revised it critically for important intellectual content. All authors read and approved the final manuscript.

## Declarations

This article has been published as part of *BMC Pregnancy and Childbirth* Volume 13 Supplement 1, 2013: Preterm Birth: Interdisciplinary Research from the Preterm Birth and Healthy Outcomes Team (PreHOT). The full contents of the supplement are available online at http://www.biomedcentral.com/bmcpregnancychildbirth/supplements/13/S1.

All of the publication fees will be funded by the Preterm Birth and Healthy Outcomes Team Interdisciplinary Team Grant (#200700595) from Alberta Innovates - Health Solutions, formerly the Alberta Heritage Foundation for Medical Research.
